# A better but persistently low health status in women with fibromyalgia during the COVID-19 pandemic: a repeated cross-sectional data analysis

**DOI:** 10.1007/s00296-022-05127-y

**Published:** 2022-04-12

**Authors:** Tim Y. Koppert, Henriët van Middendorp, Rinie Geenen

**Affiliations:** 1 Institute of Psychology, Bachelor Education Unit, Leiden University, Wassenaarseweg 52, 2333 AK Leiden, The Netherlands; 2 Institute of Psychology, Health, Medical, and Neuropsychology Unit, Leiden University, Leiden, The Netherlands; 3 Department of Psychology, Utrecht University, Utrecht, The Netherlands; 4 Altrecht Psychosomatic Medicine, Zeist, The Netherlands

**Keywords:** COVID-19 pandemic, Fatigue, Fibromyalgia, Mental health, Pain, Physical health

## Abstract

Multiple overlapping and complementary theoretical arguments suggest that the COVID-19 pandemic could worsen health in fibromyalgia. The aim of this study was to determine mental and physical health in women with fibromyalgia before and during the pandemic. In a 3-sample, repeated cross-sectional design, we analyzed questionnaire data from Dutch women with fibromyalgia, collected in three independent samples: before the COVID-19 pandemic (2018; *n* = 142) and during the first acute (2020; *n* = 304) and prolonged (2021; *n* = 95) phases of the pandemic. Eight dimensions of mental and physical health were assessed using The RAND 36-Item Short Form Health Survey (RAND SF-36). Compared to norm group data, both before and during the pandemic, women with fibromyalgia showed high levels of fatigue and pain and low levels of general health, social functioning, physical functioning, role physical functioning (*d* > 1.2, very large effect sizes), role emotional functioning, and mental health (0.71 < *d* < 1.2, medium to large effect sizes). Contrary to theoretical expectation, levels at five health variables before vs. during the pandemic did not differ (*p* > 0.05), and levels of pain (*p* < 0.001), role physical functioning (*p* < 0.001), and physical functioning (*p* = 0.03) (0.014 ≤ *pη*^*2*^ ≤ 0.042, small effect sizes) reflected a healthier status during than before the pandemic. These findings indicate a somewhat better but persistently low health status in women with fibromyalgia during the pandemic. This suggests that the pandemic may include changed circumstances that are favorable for some women with fibromyalgia.

## Introduction

For healthy and unhealthy people, the COVID-19 pandemic may cause stress and distress by worry of getting infected, changes in daily routines and caregiving, decreased opportunities for social and leisure activities, the illness or death of family members or friends, loss of work, or financial concerns [1]. In addition, for people with a chronic condition, the disease may get worse because of delayed medical evaluations, reduced access to health services, and disrupted treatment [2]. Furthermore, symptoms such as pain and fatigue encompass mutually interacting biological, psychological and social factors [3], which suggests that they may be amplified by stress of the pandemic. Specifically in fibromyalgia, central nervous system processes such as central sensitization and loss of descending analgesic activity [4], may augment pain and other somatic symptoms in response to stress [5, 6]. All in all, there are multiple overlapping and complementary theoretical arguments to expect that COVID-19 stress may worsen mental and physical health in people with fibromyalgia.

However, this expectation that the COVID-19 pandemic might lead to lower health in people with fibromyalgia, is not consistently confirmed by research. In a qualitative study, next to exacerbation of pain and fatigue, patients also reported better quality of life [7]. In longitudinal studies [8–11] with assessments before and during the pandemic or comparing a sample during the pandemic with a historic pre-pandemic sample [12], self-reported health of patients with fibromyalgia did not differ before, during or after the lockdown [8, 10, 11]. In one study, worse health during the lockdown [9] was indicated, but in another study, health improved [12]. Also, in our study including people with fibromyalgia among other groups with persistent physical symptoms, somatic symptom severity was suggested to be lower during than before the pandemic [13].

The studies analyzing quantitative data commonly analyzed the first acute phase of the corona pandemic, were conducted in small samples (31 < *N* < 80), and reported mainly composite health scores comprising mental health, physical functioning, and symptom severity without distinguishing between these dimensions. Novel aspects of our study are that (1) it was conducted in large samples, (2) included both the acute and a later phase of the pandemic, (3) evaluated distinct dimensions of health instead of only one composite measure, and (4) evaluated health as compared to a general population norm reference group. We collected data in three separate samples of people with fibromyalgia before (2018) and at two times during the pandemic: during the first major peak (2020; acute phase) and one year later when the contamination rate and restrictive measures were again high in the Netherlands (2021; prolonged phase). The aim of the current study was to determine levels at eight dimensions of mental and physical health in people with fibromyalgia before the pandemic and during two pandemic periods. Based on theoretical grounds, worse scores during the pandemic were expected, but observations of composite scores in previous studies appear to refute this expectation. Our study might give an indication about the specific dimensions of fibromyalgia health that do and do not change during the pandemic.

## Materials and methods

### Participants

This repeated cross-sectional design included three separate online surveys in the general Dutch population. The first data collection was from November 2018 to May 2019 (year 2018, pre-pandemic). The second and third collections were from March to May during the acute (year 2020) and prolonged (year 2021) phases of the COVID-19 pandemic; these were peak periods in terms of number of (intensive care) hospitalizations and deaths due to COVID-19, and in terms of strict regulations to prevent further spread of COVID-19. In the questionnaire, respondents indicated their chronic health condition(s), including fibromyalgia. For this study, only data of women with fibromyalgia were analyzed, because the number of men was too low for reliable analyses.

### Procedure

Participants were acquired via e-mail and social media, e.g., Facebook, Instagram, LinkedIn, local internet sites, and sites of associations including patient associations for fibromyalgia. The hyperlink to the online survey on individual and group sites was shared by other individuals and groups. Participants filled out the online survey at a secure university website. They self-reported their medical conditions and diseases. Participants gave informed consent prior to inclusion in the study. An inclusion criterion for the study was adult age (≥ 18 years.). An inclusion criterion for the current analysis was a self-reported diagnosis of fibromyalgia. There were no other inclusion criteria. Data collection was anonymous; it is theoretically possible that some persons participated in more than one of the surveys. The study has been performed in accordance with the ethical standards laid down in the 1964 Declaration of Helsinki and its later amendments. The online questionnaire studies in 2018 (FETC17-120, December 5, 2017) and 2020 (FETC20-190, March 23, 2020) were approved by the Ethics Committee at Utrecht University and the study in 2021 (2021–02-16-Henriet van Middendorp-V2-2959, February 16, 2021) by the Psychology Research Ethics Committee at Leiden University, the Netherlands.

### Materials

To assess mental and physical health, we used the Dutch version [14] of the RAND 36-Item Short Form Health Survey (RAND SF-36), which measures eight dimensions of health: physical functioning, social functioning, role limitations due to physical problems (role physical), role limitations due to emotional problems (role emotional), mental health, fatigue, pain and general health perception. High scores define more favorable health. The internal consistency reliability of these dimensions was good: Cronbach’s alphas ranged from 0.79 for social functioning to 0.94 for the physical functioning dimension.

### Statistical analyses

To get an indication of the health of women with fibromyalgia as compared to normal, we calculated for each dimension the standardized mean deviation from the norm score [14]. Levels on the eight health dimensions before (2018) and during the two peak phases (2020, 2021) of the COVID-19 pandemic were compared in analyses of covariance. Age, education level and having a comorbid disease, were correlated with (at least one of) the eight scales and included as covariate in analyses. Post hoc estimated marginal means were compared between the three years using Bonferroni correction.

Although score distributions hardly deviated from normal [15], with no skewness values exceeding |1| and only the kurtosis of role emotional (− 1.7) exceeding |1|, we performed bootstrap analyses to verify the validity of the results.

## Results

Table 1 shows the characteristics of women with fibromyalgia in the pre-pandemic (2018) and pandemic (2020 and 2021) samples. Age differed between the three years: *F*(*2*,*538*) = 3.36, *p* = 0.035; these differences were marginally or not significant in post hoc tests: 2018 vs. 2020, *p* = 0.10; 2018 vs. 2021, *p* = 0.06; 2020 vs. 2021, *p* = 1.00. Neither education level (*χ*^2^(2) = 4.51, *p* = 0.11), nor having a comorbid disease (*χ*^2^(2) = 1.43, *p* = 0.49) differed between the three samples.

**Table 1:** Characteristics of women with fibromyalgia before (2018) and during the first acute (2020) and prolonged (2021) phases of the COVID-19 pandemic in the Netherlands

Year	2018 (*n* = 142)	2020 (*n* = 304)	2021 (*n* = 95)	All (*n* = 541)
Age (years)				
Mean (SD)	46.6 (10.7)	49.0 (11.5)	50.1 (10.9)	48.6 (11.3)
Range	19–69	20–80	21–79	19–80
Education level^a^, *n* (%)				
Lower	76 (54.3)	190 (62.9)	50 (53.2)	316 (59.0)
Higher	64 (45.7)	112 (37.1)	44 (46.8)	220 (41.0)
Comorbid disease, *n* (%)				
None	36 (25.4)	79 (26.0)	19 (20.0)	134 (24.8)
One or more^b^	106 (74.6)	225 (74.0)	76 (80.0)	407 (75.2)

^a^Lower: lower general secondary education (48.2%) or lower (10.8%); higher: higher general secondary education (7.4%) or higher (33.6%)

^b^Having a comorbid disease other than (overlapping) chronic fatigue syndrome, irritable bowel syndrome, somatoform disorder/somatic symptom disorder, chronic headache (not migraine), or chronic pain elsewhere in the body (not the head)

Physical and mental health scores before and during the pandemic are shown in Table 2. Both before and during the pandemic, women with fibromyalgia had medium to large mean deviation scores from the norm on role emotional functioning and mental health and, with only one exception (physical functioning in 2021), and very large deviation scores on all other health dimensions, all scores indicated worse health than norm reference values.

**Table 2:** Estimated marginal means (standard error) of mental and physical health in women with fibromyalgia before (2018) and during the acute (2020) and prolonged phases (2021) of the COVID-19 pandemic in the Netherlands

Variable	2018 (*n* = 140)	2020 (*n* = 302)	2021 (*n* = 99)	Comparison of years			Post hoc pairwise comparisons
	*M* (SE)	*M* (SE)	*M* (SE)	*F*	*p*	*pη*^2^	
Physical functioning	− 1.38 (0.08)	− 1.20 (0.05)	− 1.05 (0.10)	3.70	0.03	0.014	2018 < 2021
Social functioning	− 1.90 (0.11)	− 1.80 (0.07)	− 1.78 (0.13)	0.34	0.71	0.001	
Role physical	− 1.74 (0.09)	− 1.25 (0.06)	− 1.42 (0.11)	10.52	< 0.001	0.038	2018 < 2020
Role emotional	− 0.79 (0.12)	− 0.78 (0.08)	− 0.75 (0.14)	0.03	0.97	0.000	
Mental health	− 0.71 (0.09)	− 0.94 (0.06)	− 0.89 (0.11)	2.21	0.11	0.008	
Fatigue (reverse score)	− 1.69 (0.08)	− 1.50 (0.05)	− 1.67 (.09)	2.75	0.07	0.010	
Pain (reverse score)	− 1.74 (0.07)	− 1.37 (0.04)	− 1.45 (0.08)	11.50	< 0.001	0.042	2018 < 2020, 2021
General health	− 1.61 (0.07)	− 1.52 (0.05)	− 1.47 (0.08)	0.94	0.39	0.004	

Estimated marginal means are standardized deviation scores from the general adult population norm [14]. Lower scores indicate a worse health status

Effect sizes for estimated marginal means: |0.5|–|0.8| medium, |0.8|–|1.2| large, |1.2|–|2.0| very large [16]

Effect sizes for partial eta-squared (*pη*^2^): small = 0.01–0.06

Variables were compared while controlling for age, education level and having a comorbid disorder

Comparison of scores before and during the pandemic, showed less favorable scores pre-pandemic (2018) on pain (95% confidence interval [CI] of the standardized regression coefficient [− 0.567, − 0.188], *p* < 0.001) and role physical (95% CI [− 0.748, − 0.234], *p* < 0.001) compared to the acute pandemic phase (2020), and on pain (95% CI [− 0.541, − 0.047], *p* = 0.01) and physical functioning (95% CI [− 0.640, − 0.033], *p* = 0.02) compared to the prolonged pandemic phase (2021). Effect sizes for these differences between years were small (in between 0.014 and 0.042). No differences between the three samples were shown for the other five health dimensions.

In bootstrap analyses, differences were more pronounced and other aspects of health also showed differences between samples. The sample from 2018 reported lower physical functioning (95% confidence interval [CI] of the standardized regression coefficient [− 0.360, − 0.001], *p* = 0.048) and role physical (95% CI [− 0.682, − 0.303], *p* = 0.001) and higher fatigue (95% CI [0.021, 0.369], *p* = 0.04) and pain (95% CI [0.230, 0.524], *p* = 0.001) compared to the sample from 2020 and, apart from fatigue, also compared to the 2021 sample (95% CI [− 0.576, − 0.104], *p* = 0.009; 95% CI [− 0.583, − 0.054], *p* = 0.02; 95% CI [0.107, 0.485], *p* = 0.001, respectively). In contrast, mental wellbeing was higher in 2018 compared to 2020 (95% CI [0.021, 0.431], *p* = 0.03).

## Discussion

Both before and during the pandemic, the health of women with fibromyalgia was shown to be worse as compared to the Dutch population reference group with very large deviating scores for fatigue, pain, general health, social functioning, and (role) physical functioning, and medium to large deviating scores for role emotional functioning and mental health. Contrary to theoretical expectation, levels at five health variables before and during the pandemic did not differ, and levels of pain, role physical, and physical functioning (small differences) reflected even a healthier status in samples during than before the pandemic.

There were earlier studies indicating that mental and physical health, such as reflected in fibromyalgia severity scores, was not worse [8, 10, 11] and perhaps even better [12] during than before the pandemic. Only one study observed a lower health during the pandemic [9]. Our study was the first with a larger (> 80) sample size and the first study that differentiated between health dimensions instead of using a generic health or disease severity score. Our results clearly indicate that the health of women with fibromyalgia, on average, remains low during the pandemic, with perhaps somewhat better scores for somatic symptoms and physical functioning. The only exception was the mean mental health score that appeared lower during than before the pandemic. However, the effect size was very small and only significant in the bootstrap analysis.

Although during the pandemic the severity of fibromyalgia was also observed to worsen in a considerable part of the participants [7, 10–12], from a theoretical point of view it is unexpected that, on a group level, there was no mean change or even a positive change. This suggests that the negative impact of the COVID-19 pandemic on people with fibromyalgia is weaker than assumed. In a previous publication, we considered that some people with persistent somatic symptoms may have experienced a positive impact, for instance, because they felt less pressure from work, more social connectedness, or more recognition for their symptoms and situation during the pandemic [13]. In one study, people with fibromyalgia during the pandemic thought that their improvement was caused by beneficial effects of smart working and the opportunity to exercise more regularly [10]. In another study, some interviewed people with fibromyalgia reported that reduced social constraints allowed them to adjust the rhythms of their life to fluctuations of symptoms and that fibromyalgia stopped being a main priority in their lives [7]. These authors concluded that reducing social constraints could be a key for fibromyalgia management, where symptoms seemed to take less space in everyday life.

A strength of the current study is its time frame. People participated during the first two peak months of the virus outbreak in 2020, when COVID-19 had the most invasive consequences and during the prolonged lockdown in 2021 when many people became inpatient. Our samples did not include an equal number of participants in each year, but in every year the sample size was large enough to have small margins of error. Our study included self-reported data from people with fibromyalgia in the general population. A limitation is that we did not collect clinical data, such as current interventions (pharmacological, physical exercise, psychological) and whether treatment, such as regular physical exercise was promoted or hindered during the pandemic, which likely both may occur [10]. The results of our study do not generalize beyond the report of self-perceived health. A limitation is that our samples were convenience rather than representative. Moreover, some persons may have participated in more than one of the surveys. Because data collection was anonymous, we do not know how many. Obtaining repeated data from the same people at similar periods in the year would have yielded insight into how many people deteriorated and ameliorated. However, our results are not inconsistent with most studies measuring intra-individual changes in smaller samples of people with fibromyalgia [10–12]. We did not have perfect norm data, because the norm group is from 25 years ago and included 35% men, which may have yielded somewhat lower scores in our sample of women with fibromyalgia. Another limitation is that fibromyalgia was not confirmed by clinical assessment. Finally, considering that during the pandemic similar findings were found in European studies [10–12] and that our results deviated from deterioration observed in Mexican people with fibromyalgia [9], suggests that our data are at best generalizable to women with fibromyalgia in Western European countries.

## Conclusions

Women with fibromyalgia have, on average, a low level of mental and physical health irrespective of the COVID-19 pandemic. Our findings tentatively indicate that mean health levels do not further deteriorate during the pandemic and that somatic symptoms and physical functioning may even be better. This suggests that the pandemic may include changed circumstances that are favorable for at least part of the women with fibromyalgia.

## Open data sharing statement

The dataset analyzed during the current study is available from the corresponding author on reasonable request.
